# Elicitor-mediated enhancement of secondary metabolites in plant species: a review

**DOI:** 10.3389/fpls.2025.1706600

**Published:** 2025-10-22

**Authors:** Abdul Razzaq, Muhammad Mubashar Zafar, Aima Ali, Lubna Ihsan, Fariha Qadir, M. Nasir Khan, Yingying Zhang, Linmao Gao, Hanqing Cong, Rashid Iqbal, Xuefei Jiang, Fei Qiao

**Affiliations:** ^1^ Tropical Crops Genetic Resources Institute, Chinese Academy of Tropical Agricultural Sciences, Haikou, China; ^2^ Institute of Molecular Biology and Biotechnology, The University of Lahore, Lahore, Pakistan; ^3^ National Key Laboratory for Tropical Crop Breeding, Sanya, China; ^4^ Key Laboratory of Biology and Cultivation of Herb Medicine (Haikou), Ministry of Agriculture and Rural Affairs, Haikou, China; ^5^ Sanya Institute of Breeding and Multiplication/School of Tropical Agriculture and Forestry, Hainan University, Sanya, China; ^6^ Department of Plant Breeding and Genetics, University of Agriculture Faisalabad, Faisalabad, Pakistan; ^7^ Renewable Energy and Environmental Technology Center, University of Tabuk, Tabuk, Saudi Arabia; ^8^ College of Life Science and Technology, Huazhong Agricultural University, Wuhan, Hubei, China; ^9^ Key Laboratory of Tropical Crops Germplasm Resources Genetic Improvement and Innovation of Hainan Province, Haikou, China; ^10^ Department of Agronomy, Faculty of Agriculture and Environment, The Islamia University of Bahawalpur, Bahawalpur, Pakistan; ^11^ Department of Life Sciences, Western Caspian University, Baku, Azerbaijan

**Keywords:** secondary metabolites, elicitors, biosynthesis, alkaloids, plant metabolites

## Abstract

Plant metabolites play a vital role in a plant’s defense system. Plant metabolites are extensively studied for their therapeutic values. Plant therapeutic values are attributed based on the magnitude of metabolites. Among all the metabolites, secondary metabolites are considered to have more potential. Different medicinal plants like *Cephalotaxus* contain therapeutically valuable bioactive alkaloids. The pharmaceutical relevance of secondary metabolites has been well recognized, but low accumulation and convoluted biosynthetic mechanism hamper their industrial production. Elicitors, both biotic and abiotic, have emerged as effective strategies to enhance metabolite biosynthesis by triggering plant defense signaling pathways. Chemical agents like salicylic acid, methyl jasmonate, nitric oxide, and heavy metals, along with physical factors such as ultraviolet radiation, salinity, and osmotic stress, significantly increase secondary metabolite production. Similarly, microbial extracts, polysaccharides, and polyamines serve as potent biotic elicitors. Synergistic combinations, particularly sodium fluoride with methyl jasmonate, have shown remarkable success in boosting *Cephalotaxus* alkaloid yields. Advances in elicitor-mediated interventions, coupled with omics, nanotechnology, and CRISPR-based bioprocessing, promise sustainable and scalable production systems. This review highlights the mechanisms, case studies, challenges, and prospects of elicitor applications, emphasizing their transformative role in bridging traditional medicinal plants with modern pharmaceutical needs.

## 1 Introduction

Plants synthesize a wide range of small organic compounds known as metabolites, which are vital for their survival. These molecules are functionally considered into three primary groups. Primary metabolites (PM), such as organic acids, amino acids, and nucleotides, are directly responsible for fundamental growth and development. Secondary metabolites (SM) mainly interfere with the plant’s interactions with its environment, for example, defense or attraction. Tertiary metabolites are crucial modulatory compounds that control internal physiological processes, including the synthesis of other metabolites. Secondary metabolites (SMs) are structurally varied compounds that play an important role in allowing plants to defend themselves and acclimate themselves to abiotic and biotic stresses. They are classified based on either their chemical composition or biosynthetic pathway (Zulfiqar & Ashraf, 2021).

Plant secondary metabolites are small organic molecules with a molecular mass of 3000 Da (Tariq et al., 2023). The therapeutic value of medicinal plants is mainly attributed to their varied array of SMs. Plants produce these valuable compounds as an adaptive response to environmental challenges. The synthesis of SM is influenced by many factors, such as genetics, climate, and nutrient availability. When plants confront stresses such as pathogens, drought, salinity, or extreme temperatures, receptors on their plasma membrane perceive these threats and activate a signaling cascade that eventually stimulates the synthesis of SMs (Wani et al., 2023).

The major classes of plant SMs include alkaloids (harringtonine and homoharringtonine), terpenes, flavonoids, phenolics, and steroids. These compounds execute perilous ecological functions, from deterring herbivores and modulating symbiotic relationships to varying the structure of soil microbial communities. Meanwhile, synthesis of SM is persuaded merely by biotic or abiotic stressors. The naturally occurring SMs are normally found in low quantities (Uchimiya, 2022). They assist in various defensive roles, such as providing protection from UV radiation, acting as antioxidants, inhibiting enzymes, and producing pigments. These secondary compounds are synthesized from primary metabolites through various biochemical pathways and are broadly categorized as nitrogenous or non-nitrogenous. About 30,000 terpenoids, 21,000 alkaloids, and 8,000 phenolic compounds have been identified so far. Nitrogenous compounds include amines, amino acids, glucosinolates, non-proteinogenic compounds, cyanogenic glycosides, and alkaloids. Alkaloids themselves are a diverse group that are classified into proto, cyclopeptide, polyamine, true, and pseudo alkaloids. They are biosynthesized from amino acids as their primary building blocks (Mabou and Yossa, 2021). Non-nitrogenous secondary metabolites include polyacetylenes and phenolics, and are categorized into four main groups, which include arylpyrones, styrylpyrones, terpenoids, and polyketides. Phenolic compounds are synthesized through the shikimic acid and malonate or acetate pathways and are largely divided into phenolic acids and phenylpropanoids. The last group is highly multifarious, consisting of eight flavonoids, lignans, coumarins, and lignin. Flavonoids themselves are further classified into subgroups, including flavonols, flavones, isoflavones, flavanones, and anthocyanins. Another important category, terpenoids, is all derived from the five-carbon precursor isopentenyl diphosphate (IPP), which is produced by either the mevalonate pathway or the methylerythritol phosphate pathway. Terpenoids are steadily classified based on the number of isoprene units they contain, resulting in groups such as monoterpenes, sesquiterpenes, etc (Naikoo et al., 2019). Stress-sensitive crops often accumulate elevated levels of valued SMs, especially when visible to specific elicitors or signaling molecules. These bioactive compounds, such as alkaloids, polyphenols, glycosides, and phytosterols, are highly required by the pharmaceutical and nutraceutical industries. The practice of elicitation, which uses pharmacological agents to activate this natural defense response, has emerged as a promising field of study. Elicitors function by triggering specific transcription factors, which in turn stimulate key genes, thereby activating the metabolic pathways responsible for the synthesis of these valuable compounds. This approach offers substantial potential for significantly increasing yield and generating substantial financial benefits (Jain et al., 2024). The global market for plant-derived secondary metabolites is substantial, valued at approximately US$30 billion annually, underscoring their immense economic and therapeutic significance. However, supply shortages remain a critical bottleneck, particularly as an estimated 70% of anticancer drugs are of natural plant origin, highlighting an urgent need for enhanced and sustainable production strategies (Newman and Cragg, 2020).

The objective of this review is to comprehensively evaluate the role of biotic and abiotic elicitors in enhancing the biosynthesis of secondary metabolites in medicinal plants, with a particular focus on *Cephalotaxus* species producing therapeutically important alkaloids such as harringtonine (HT) and homoharringtonine (HHT). It aims to analyze the types, mechanisms, and synergistic effects of elicitors, highlight case studies demonstrating their efficiency, and explore future strategies—including biotechnological, omics-based, and nanotechnology approaches—for sustainable and scalable production of bioactive compounds for pharmaceutical applications.

## 2 Elicitors: types and mechanisms

The elicitors are molecules that activate the synthesis of metabolites, for example, phytoalexins. Elicitors are agents that adjust the amount and composition of particular compounds in plants, such as secondary metabolites (SMs). These metabolites are shaped by biotic and abiotic stresses. SMs play a vital role in permitting plants to acclimatize and flourish under challenging conditions (Humbal and Pathak, 2023). The plant kingdom is home to a vast array of SMs, which differ in structure and composition. Although they are not vital for plant growth and function, these compounds serve as repellents for herbivores and lure pollinators by facilitating the modifications in pigmentation and aroma of parts of plants. Nevertheless, their use in pharmaceuticals, drug development, nutraceuticals, agriculture-based products, and the food industry has made them economically valuable. Therefore, numerous agronomic and biotechnological techniques have been employed to boost their availability, generation, and storage in plants (Ali, 2021b). Elicitation is a powerful and effective technique in plant biotechnology to initiate the manufacturing of novel SMs and enhance biomass yield and metabolite accumulation in *in vitro* cells. In the plant system, elicitors aid in elevating the production of particular secondary metabolites. In turn, these SM regulate the developmental processes, stress tolerance, survivability, productivity, and signal transduction pathways. Several factors, including the type of elicitor, treatment duration, dosage, and culture type, influence the effectiveness of elicitation. Studies have confirmed enhanced synthesis of specific SMs in hairy root and cell suspension cultures (Zheng et al., 2023). The chemical compounds and alkaloids derived from *Cephaloatxus* species have been used for leukemia patients. Different methods have been employed to increase the yield of alkaloids such as harringtonine and homoharringtonine, but achieving industrially viable yields remains a significant challenge. However, bridging the gap of traditional knowledge to modern therapies, elicitors are playing a vital role in achieving the required yield of alkaloids. The following are the potential elicitors that could be used in studies to optimize their yield.

## 3 Abiotic elicitors

### 3.1 Chemical elicitors

Chemical elicitors simulate stress conditions in medicinal plants, enhancing the biosynthetic effects of therapeutic SMs such as flavonoids and alkaloids. Chemical elicitors include salicylic acid, methyl jasmonates, nitric oxide donors, and heavy metals.

#### 3.1.1 Salicylic acid

Salicylic acid (SA) is a multifunctional, and major plant derived phenolic that governs numerous mechanisms important for plant development ranging from germination of seeds, pigment biosynthesis, modulation of enzyme activity, ethylene synthesis, stomatal behavior, heat generation, photosynthetic efficiency, nutrient uptake, floral initiation, membrane stability, nodulation in legumes, ultimately shaping the plant’s overall health and development (Rivas-San Vicente and Plasencia, 2011). SA exhibits hormone-like properties, and it has been employed in a variety of plant models, both *in vitro* and *in vivo*, to assess its regulatory effects on biosynthesis and accumulation of SMs by upregulating different pathways, such as the MEP/MVA route. The MEP/MVA pathway is important for harringtonine precursors. The incorporation of SA in the culture medium and transient exposure of plant cultures have shown significant amplification of secondary metabolite biosynthesis and accumulation (Ali, 2021b). SA acts as a powerful elicitor in medicinal plants to facilitate the biosynthesis of terpenoids, phenolics, and nitrogen-based secondary metabolites. Studies have revealed that numerous medicinal plants, when treated with SA, increase the content of triterpenoids, camphor, cincole, farnesol, and isochiapin B sesquiterpenoids on administration into *Panax ginseng* adventitious and a notable upsurge of triterpenoids in *Centella asiatica* (Buraphaka and Putalun, 2020). The established role of SA in other species suggests it is a solid candidate for testing precursor and pathway modulation in *Cephalotaxus*.

#### 3.1.2 Jasmonic acid & methyl jasmonate

As abiotic elicitors, JA and its derivatives modulate the physiology of plants as well as strengthen their defense system by promoting the synthesis of pivotal SMs, including essential oils, phenolics, terpenes, and alkaloids (Ali, 2021a). These elicitors maintain distinctive roles across plant lineages, whether gymnosperms and angiosperms, by interacting with receptors of the plasma membrane, sparking a defense cascade involving NOS/ROS accumulation and production of protective oxidative stress enzymes (Ho et al., 2020). The mechanism of JA and MeJA is well studied in non-*Cephalotaxus* species; however, significant results from cell culture of *Cephalotaxus* form an exception (Li, 2014), explaining the synergetic effect of NaF and methyl jasmonate (MJ) in *C. mannii* cultures, which resulted in a significant elevation in biosynthesis and release of harringtonine and homoharringtonine. The administration of NaF results in enhanced G6PDH, indicating stimulation of primary metabolism; however, MJ specifically increases activation of PAL enzyme, suggesting a surge in secondary metabolite biosynthesis. It was evident that synergistic enhancement of NaF and MJ significantly raised harringtonine to 4.8-fold and detectable homoharringtonine. Another study explored the changes induced by treatment of MeJA on steroids and triterpenoids biosynthesis, assessed in two hairy root lines of Taxus x media, KT and ATMA. In response to MeJA, ATMA, having lower baseline metabolite levels, exhibited an increase in steroid levels, whereas KT showed a drop. Despite the differences in both lines, they share a response in increased sterol glycoside content, elevated stigmasterol, and a rise in phenolics and triterpenoids (Sykłowska-Baranek et al., 2022).

#### 3.1.3 Nitric oxide

Nitric oxide has emerged as a key regulator in the elicitation of plant defense mechanisms and SMs production. When exposed to stress conditions, nitric oxide (NO) serves as a central signaling agent and enhances the production of SMs in plants. Nitric oxide (NO), commonly secreted in medicinal plant cultures, regulates signaling pathways involved in growth, defense, programmed cell death, and stress response, leading to heightened secondary metabolite biosynthesis (Zhang et al., 2012). It also serves as a protective signal, reinforcing antioxidant pathways, strengthening redox balance, ensuring osmotic and water homeostasis, controlling ion fluxes, sustaining photosynthesis, and thereby maintaining plant physiology. By mediating post-translational modifications of proteins, NO influences the activity of enzymes and transcription factors, and controls the expression of stress genes (Allagulova et al., 2023). Several studies have demonstrated that exogenous applications of sodium nitroprusside (SNP), a nitric oxide donor, have proven effective in inducing the production of SMs, including marjoram essential oil, phenolics, and flavonoids in *Echinacea purpurea*, Taxol, and *Taxus yunnanensis* cultures, and artemisinin in *Artemisia annua* (Farouk and Al-Huqail, 2020). SNP has been employed as a chemical elicitor to stimulate hypocrellin biosynthesis, derived from the fungal perylenequinone pigments of *Shiraia*, which exhibits strong anticancer activity in photodynamic therapy (Ma et al., 2021).

#### 3.1.4 Heavy metals

Although heavy metals are toxic to most crops, in medicinal and aromatic plants, heavy metals can act as abiotic elicitors, as these plants exploit heavy metal stress as a trigger to enhance biosynthesis of valuable SMs. A complex network of coordinated physiological, biochemical, morphological, and genetic adaptations is acquired by the plants to withstand this stress. Heavy metals initiate ROS generation by disrupting cellular enzymes by replacing vital ions, and cause oxidative injury in plant cells. Plants deploy a combination of antioxidant enzymes like catalase and superoxide dismutase, and non-enzymatic molecules such as vitamins, phenolics, and glutathione to mitigate ROS-induced stress. It is seen that controlled exposure of medicinal plants to these heavy metals could be harnessed to enhance the accumulation of therapeutic SMs, creating opportunities for optimized cultivation (Maleki et al., 2017). Heavy metals like silver (Ag), cadmium (Cd), and copper (Cu) act as abiotic elicitors in medicinal plants, stimulating the production of phenolic compounds, enhancing their defense through chelation and antioxidant activities (Thakur et al., 2019). Researchers also evaluated that Ag^+^ and Cd²^+^ supplementation modulates bioactive flavonoids, phenolic acids, and oxidative stress response in *in vitro* shoot cultures of *Dracocephalum ruyschiana*, valuable for its therapeutic potential in Magnolia, without any adverse effects on growth, underscores the potential for testing similar approaches in *Cephalotaxus*, but direct suggestion for increasing HT and HHT have not yet been reported (Weremczuk-Jeżyna et al., 2024). Another study reported that application of CuO nanoparticles resulted in elevated levels of vital SMs like purpurin and alizarin, and improved antioxidant activity in plants. This study confirms that beyond the threshold level, CuO ceased to enhance metabolism and instead imposed toxic stress; however, low doses were an effective *in vitro* tool to boost bioactive compound synthesis, offering valuable implications for both pharmaceutical and textile applications (Prasad et al., 2025).

However, several other chemical elicitors, such as trehalose, have been used and are involved in various biosynthesis pathways to produce secondary metabolites in either way. Trehalose-6-phosphate synthase (TPS) is encoded by 7 *ClTPS* genes, located in 7 different chromosomes of the watermelon genome. Expression analysis by qRT-PCR revealed that osmotic stress could upregulate the expression of all ClTPS family members and promote trehalose accumulation in watermelon cells accordingly. Exogenous methyl jasmonate (MeJA), ethephon (ETH), abscisic acid (ABA), or salicylic acid (SA) induced trehalose accumulation, with MeJA being the most effective treatment (Zhu et al., 2022). It is suggested that osmotic stress-induced glycinebetaine biosynthesis occurs via JA signal transduction and not only plays a key role in osmotic stress resistance but also contributes to osmotic stress hardening and secondary metabolite production (Xu et al., 2018).

### 3.2 Physical elicitors

#### 3.2.1 Ultraviolet radiation

UV radiation is emerging as a significant environmental factor that promotes the synthesis of SMs in plants. The ultraviolet spectrum is segmented into UV-A (315nm - 400nm), UV-B (280nm - 315nm), and UV-C (below 280nm). Recent research reveals that low levels of UV-B radiation exposure are particularly effective in boosting the levels of phenolic compounds like glycosylates and flavonoids in plants (Rizi et al., 2021). Increased antioxidant activity and phenolic production under the influence of UV-B were most prominent in young leaves of *Salvia verticillata*, while reducing its biomass and chlorophyll content. Analysis of gene expression uncovers that UV-B stimulates phenylpropanoid pathway genes, particularly boosting the transcription of tyrosine aminotransferase (TAT), rosmarinic acid synthase (RAS), and phenylalanine ammonia-lyase (PAL), with peak induction in young leaves after 10 days (Rizi et al., 2021). Another study carried out by Zhong et al. (2019) explored the SMs production in *Catharanthus roseus* by UV-B, employing phosphoproteomic and metabolomic approaches. As a response to UV-B, ATP levels were elevated and upregulated calcium-mediated proteins such as calcium-dependent kinase, calmodulin, and heat shock proteins, along with significant alterations in proteins engaged in cell signaling, glycolytic pathways, energy metabolism, post-translational modification, and ROS detoxification. Metabolomic profiling showed a rise in aromatic amino acids, pentose sugars, and phenylpropanoid derivatives. Hence, it was concluded that UV-B exposure played a key role in enhanced SMs production and redox-regulated pathways in *C. roseus.* Leveraging UV-B as an elicitor could potentially enhance the biosynthesis of valuable alkaloids and act as a novel strategy for optimizing high harringtonine yield in *Cephalotaxus* species, as evidenced in other medicinal plants under controlled conditions.

#### 3.2.2 Salinity

Salt stress alters various physiological and metabolic pathways in plants, triggering adaptive mechanisms to mitigate ionic, osmotic, and oxidative stress. Using controlled salinity as an elicitor offers a resourceful way to enhance SMs accumulation without compromising plant vitality. However, in-depth analysis is required to elucidate the metabolic pathways and improve production efficiency (Halder et al., 2019). One of the studies showed that hydroponically cultivated kale and Chinese cabbage, white cabbage, when exposed to moderate salinity levels (50 -100mM NaCl), improved the accumulation of health-beneficial compounds such as glucosinolates, phenolics, and chlorophyll, while largely preserving their nutritional mineral content. The results indicated that Chinese cabbage experienced significant stress; in contrast, kale remained tolerant to salt stress. However, the levels of important nutrients like potassium and calcium were successfully maintained in both of the vegetables. These findings imply the potential of leveraging regulated salt stress to boost the health benefits and enhance the phytochemical profile of plants cultivated in salty soils (Šamec et al., 2021). The application of NaCl in *in vitro* cell culture conditions demonstrated the positive effects by biosynthesis of anti-cancerous compounds, vinblastine and vincristine, in the cultured shoots and leaves of *Catharanthus* roseus L (Fatima et al., 2015). NaCl-induced treatment to callus cultures of *Mentha longifolia* was reported to enhance the rosmarinic acid and phenolic contents (EL-Shennawy et al., 2017). The standardization of elicitors depends on optimization of metabolite level and type of salt, for instance, applying 250mM NaCl raised sitosterol levels in *Nitraria tangutorum* cell suspensions (Ni et al., 2015).

Research on *Ocimum basilicum* (basil) reported that when exposed to salt stress, it stimulated the accumulation of certain phenolic acids such as caftaric acid, caffeic acid derivatives, feruloyl tartaric acid, and cinnamyl malic acid; however, there is a decline in chicoric acid levels (Scagel et al., 2019). A study examined the effects of varying concentrations of NaCl (0-200mM) on *Inula crithmoides* (golden samphire) shoot cultures, for a period of 4 weeks, to determine the responses in growth, physiology, and secondary metabolite content. Moderate salinity enhanced the photosynthetic performance, oxidative stress tolerance, and increased reserves of proteins, proline, and soluble sugars. Salt elicitation promoted the phenolic compounds, including gentisic acid, chlorogenic acid, naringenin-7-O-glucoside, and luteolin-7-O-glucoside, also supporting its neutraceutical potential and wellness in saline environments (Rodrigues et al., 2024). Salinity stress can trigger metabolite synthesis in many plants, and they often counter salt stress by accumulating bioactive compounds like tannins, alkaloids, flavonoids, phenols, anthocyanins, proline, and saponins, but effects differ strongly across species and plant parts. In *Origanum majorana*, treatment with NaCl suppressed trans-sabinene hydrate levels; however, salinity stress exhibited metabolite accumulation in some plants, like flavonoid in *Ginkgo biloba*, flavonoid and phenol in *Cucumis sativus* and *Solanum lycopersicum*, phenol, flavonoid, flavones, anthocyanin in *Carthamus tinctorius*, proline, flavonoids and saponins in *Plantago ovata*, anthocyanins in *Myrica pensylvanica*, and tannin in *Achillea fragratissima* (Humbal and Pathak, 2023).

#### 3.2.3 Osmotic stress

Osmotic stress is often triggered by salinity, drought, and freezing, contributing as a serious abiotic stress factor that hampers water availability, consequently resulting in water scarcity and ultimately drastically declining plants’ health and productivity. Besides its effects on the growth of plants, it also influences the regulation of SMs synthesis, with evidence in certain plants when exposed to such stress conditions (Upadhyaya et al., 2013). For example, polyethylene glycol (PEG) is widely harnessed as a non-toxic inducer of osmotic stress without causing plasmolysis; however, it can impair photosynthetic components like photosystems, pigments, and electron transport chains, ultimately diminishing photosynthetic activity (Zhang and Shi, 2018). PEG molecules of high molecular weight are unable to penetrate the cell wall matrix. When they are applied in culture medium, they cause reduced oxygen diffusion, hence resulting in the simulation of drought conditions in plants (Martínez-Santos et al., 2021). A study indicated valuable therapeutic properties of Rutin, an SMs derived from *Ruta graveolens*, and approaches to improve its bioactive synthesis using mannitol and PEG to induce osmotic stress in cell suspension cultures. The findings from the *in vitro* analysis suggested that the best results were gained by mannitol elicitation, as it led to peak cell growth (250mg/day) at 50 g/L and maximum Rutin synthesis level (60.15mg/L) at 100 g/L, slightly reducing yield of 45.1mg/L at 150 g/L (Mahood, 2021). The application of osmotic stress not only increased secondary metabolite accumulation, raising capsaicin in *Capsicum chinensis* cultures, but also treatment with proline and PEG promoted the synthesis of steviol glycosides in *Stevia rebaudiana* and callus suspension cultures (Gupta and Jain, 2022). Cultivation of *A. montana* hairy roots on MS medium containing 3-5% sucrose displayed increased biomass production, and results from GC-MS confirmed a diverse range of metabolites, including phenolic acids, flavones, fatty acids, amino acids, organic acids, sugar alcohols, and hydrocarbons (Petrova et al., 2015). Treatment with sorbitol indicated elevated levels of malanodialdehyde and hydrogen peroxide, triggering oxidative stress in *A. annua* cell suspension culture; however, its combination with coronatine synergistically upregulated artemisinin biosynthetic gene expression and boosted artemisinin content in all tested doses (Salehi et al., 2019). By applying eight different mannitol or sorbitol treatments, a successful protocol was established for the slow-growth preservation of *A. montana* shoot cultures *in vitro*, maintaining its quality for up to six months (Petrova et al., 2021). Abiotic elicitors augmenting plant secondary metabolites in different plant species is given in **Table 1**.

**Table 1:** Abiotic elicitors augment plant growth and the production of secondary metabolites in different plant species.

Elicitor	Plant species	Nature of culture	Compounds	Reference
Jasmonic acid	*Bacopa monnieri*	Shoot	Bacoside A	(Sharma et al., 2015)
	*Plumbago indica*	Hairy root	Plumbagin	(Gangopadhyay et al., 2011)
	*Plumbago rosea*	Cell suspension	Plumbagin	(Silja et al., 2014)
Methyl jasmonate	*Salvia miltiorrhiza*	Hairy root	Tanshinone	(Hao et al., 2015)
	*Perovskia abrotanoides*	Adventitious roots	Cryptotanshinone and tanshinone IIA	(Zaker et al., 2015)
	*Salvia officinalis*	Shoot	Diterpenoid	(Grzegorczyk and Wysokinska, 2009)
	*Bacopa monnieri*	Shoot	Bacoside	(Sharma et al., 2013)
	*Gymnema sylvestre*	Cell suspension	Gymnemic acid	(Chodisetti et al., 2015)
Gibberellic acid	*Salvia miltiorrhiza*	Hairy root	Tanshinones	(Yang et al., 2024)
	*Echinacea pupurea*	Hairy root	Caffeic acid derivatives	(Abbasi et al., 2012)
Salicylic acid	*Salvia miltiorrhiza*	Hairy root	Tanshinone	(Hao et al., 2015)
	*Vitis vinifera*	Cell suspension	Stilbene	(Hao et al., 2015)
	*Digitalis purpurea*	Shoot	Digitoxin	(Patil et al., 2013)
	*Withania somnifera*	Hairy root	Withanolide A, withanone, and withaferin A	(Sivanandhan et al., 2013)
	*Glycyrrhiza uralensis*	Adventitious root	Glycyrrhizic acid	(Li et al., 2016)
NaF+MJ	*Cephalotaxus* sp.	Cell culture	Cephalotaxine	(Li, 2014)

## 4 Biotic elicitors

Biotic elicitors are biologically obtained compounds produced by disease-causing agents or the host plant itself. These comprise damage-associated molecular patterns (DAMPs), which act as signaling molecules. Biotic elicitors can be classified into two main groups, i.e., (1) microorganism-derived and (2) polysaccharide-based compounds (Bhaskar et al., 2022). As endogenous elicitors, DAMPs generally act as menace signals, triggering innate defense mechanisms within the apoplast (Cai et al., 2013). Exogenous elicitors include bacteria, viruses, herbivore incursions, and fungi, which trigger plants to produce wary compounds at the site of attack. These elicitors act through receptor-mediated mechanisms, modulating enzymes or ion channels. Pathogens like bacteria and fungi release some specific digestive enzymes (e.g., polygalacturonase), cell wall fragments, and peptides that break down plant tissue barriers, such as the middle lamella. Furthermore, gene-specific elicitors derived from pathogen virulence genes further influence plant responses (Kawauchi et al., 2016).

### 4.1 Microbial (fungal/bacterial extracts) elicitors

Biotic stress affecting crop species leads to a decrease in yield and quality. As a result of pathogen attacks, plants produce defensive complexes that help alleviate disease effects. They coincide with varied microbial communities, playing a key role in plant health by increasing nutrient uptake, variable hormones, and providing defense against various pathogens. One key microbial mechanism in biocontrol is induced systemic resistance (ISR). Some helpful microbes release elicitors in the rhizosphere that are perceived by plant roots, activating improved defense mechanisms and refining resistance to phytopathogens (Kour et al., 2024). The interface between microbes and medicinal plants is an intricate and dynamic process that plays a vital role in SMs production. Microbial inocula, which include bacteria, fungi, yeasts, algae, and viruses, can meaningfully improve the synthesis of such bioactive compounds. Though SMs naturally make up less than 1% of a plant’s dry weight, their production is affected by the plant’s physical state and developmental stage. Microbes act as biotic stimulants that trigger plants to control their metabolic pathways, which can lead to augmented SMs synthesis. This response helps plants defend against biotic and abiotic stresses (Kumari et al., 2024).

Some plants activate their biochemical pathway in response to the external stresses that increase SMs production. These stress responses can be activated by several elicitors, including endophytic fungi, which share a long-standing synergetic relationship with medicinal plants. Fungal elicitors quickly persuade specific gene expression, resulting in the activation of metabolic pathways that increase the accumulation of bioactive compounds (Zhai et al., 2017). Some researchers have demonstrated that fungal elicitors are extremely active for increasing SMs production in plant cell cultures and stimulating growth in hairy roots (Takeuchi et al., 2013). Few studies also explore signal crosstalk, gene expression, enzyme initiation, and practical applications. Fungal elicitors efficiently excite SMs production by imitating natural plant defense responses against pathogens (Chen et al., 2015). Endophytic fungal elicitors quickly and surely excite specific gene expression in medicinal plants, beginning dedicated secondary metabolic pathways that drive an extensive increase in active compound accumulation (Zhai et al., 2017).

Elicitation with five bacterial and five fungal strains in root cultures of *Taverniera cuneifolia* (Roth), reported to boost the synthesis of important secondary metabolites, particularly glycyrrhizic acid (GA), a compound with known medicinal properties. Within the fungal elicitors, GA production was maximum with *Mucor hiemalis* (4.90 ± 0.10 mg/g); in contrast, *Aspergillus tenuis* yielded the lowest levels of GA (1.50 ± 0.07 mg/g). Among the bacterial elicitors, GA production peaked with *Rhizobium leguminosarum* (6.37 ± 0.41mg/g), while *Agrobacterium tumefaciens* showed minimum induction (1.46 ± 0.06 mg/g) (Awad et al., 2014).

Elicitation with 2.5% *B. subtilis* in cell suspension cultures of *Panax sikkimensis*, together with *Serratia marcescens* showed enhanced ginsenoside synthesis (Biswas et al., 2018). The greatest induction of ginsenosides and anthocyanins was attained with the elicitation of culture filtrates of *Trichoderma harzianum* and *Trichoderma atroviride* in *P. sikkimensis* cell suspension (Biswas et al., 2018). Elicitation with both Gram-positive and Gram-negative bacterial strains is used to achieve ginsenoside production in *Panax ginseng*, however, Gram-negative bacteria, including *Bradyrhizobium, Mesorhizibium, Azotobacter* outperform Gram-positive ones, such as *Leuconostoc, Bacillus, and Lactobacillus*, and exert stronger elicitation effects (Le et al., 2018). Moreover, Diosgenin production in *Helicteres isora* L. suspension cultures was seen to be promoted by filtrates derived from *B. subtilis* and *E.coli* as elicitors (Shaikh et al., 2020). In one of the medicinal shrubs *Oldenlandia umbellata* L., an important secondary metabolite dye is effectively enhanced when cultures are treated with fungal elicitors such as *Mucor prayagensis*, *Trichoderma viride*, and *Aspergillus niger* (Le et al., 2018).

### 4.2 Polysaccharides (chitosan, yeast extract, bacterial lysates)

Endophytic fungi are microorganisms that live inside healthy plant tissues during part or all of their life cycle without producing substantial damage to the host. They create a symbiotic association with the plant, sustaining an active balance that often benefits both organisms (Qin et al., 2024). Certain endophytic fungi improve the production of plant secondary metabolites by secreting polysaccharide elicitors. Meanwhile, hairy roots, genetically stable, fast-growing structures that produce SMs at levels similar to whole plants, serve as an exceptional source of bioactive compounds. As a result of these advantages, hairy root expertise has emerged as a promising approach for enlightening therapeutic plant quality and efficiently gaining valuable SMs (Wang et al., 2023).

In *Pueraria candollei*, isoflavonoid yield increases when treated with yeast extract. In *Salvia miltiorrhiza* Bunge cell cultures, it serves to elicit tanshinone production, and in cell suspension cultures of *Catharanthus roseus*, it is also used as an elicitor for the biosynthesis of vinblastine, vincristine, and other alkaloids (Maqsood and Abdul, 2017). In medicinal plants, chitosan is employed as an elicitor to enhance the biosynthesis of secondary metabolite production, including Withaferin-A in *Withania somnifera*, plumbagin in *Plumbago rosea*, and artemisinin in *Artemisia annua* (Thilip et al., 2019). It was seen that dextran and laminarin (bacterial-derived polysacchrides), when applied to wounds of *Solanum lycopersicum* infected with *B. cinerea*, increased the production of phenylpropanoid and flavonoid levels markedly (Lu et al., 2019). One of the studies reported that application of yeast extract and chitosan boosted growth-related traits, essential oil content, and levels of bioactive metabolites in *Lippia alba.* The highest efficacy was seen with chitosan at a concentration of 4 g/L, which promoted plant growth, trichome density, and multiple novel metabolites, whereas yeast extract triggered a single metabolite (Silva-Santos et al., 2023). In *Fagopyrum tataricum* hairy root cultures, application of yeast-derived polysaccharides induced the phenylpropanoid pathway, leading to increased rutin and quercetin production and enhanced root development, with flavonoid content increased up to 2.1-fold at 200mg/L (Halder et al., 2019).

### 4.3 Hormonal and chemical elicitors

#### 4.3.1 Polyamines

Polyamines are small, positively charged biomolecules that play vital roles in plant growth, development, and stress adaptation. Their levels in plants change vigorously in adaptations to environmental signals, hormonal indications, and developmental stages. Researchers are trying to investigate the downstream molecular mechanisms through which polyamines function, as well as signaling pathways and subsequent control procedures. Recent reviews have summarized key characteristics of polyamine-mediated signaling and their physiological effects in plants (Napieraj et al., 2023). The key polyamines in plants are putrescine, spermidine, and spermine, which play dynamic roles in cell division, organ development, and stress resilience. Putrescine is the simplest polyamine and is formed from ornithine or arginine decarboxylation and accumulates in amounts associated with active cell propagation. Beyond regulating gene expression and protein synthesis, polyamines contribute to enzyme stability and cellular homeostasis (Mustafavi et al., 2018). Few studies have indicated that polyamines act as defensive and signaling molecules, increasing plant stress tolerance by binding to biological macromolecules, stabilizing cell membranes, regulating reactive oxygen species, and enhancing the production of osmoregulators like proline under abiotic stress conditions (Shao et al., 2022).

One of the studies reported that when leaves of *O. basilicum* (basil) were sprayed with polyamines such as spermidine, spermine, and putrescine at a concentration of about 100mg/L, it boosted the yield of essential oils (Danaee and Abdossi, 2019). Research was conducted using cell cultures from *Hybanthus enneaspermus* for sustainable L-DOPA production. Precursor feeding, mainly with tyrosine, increased yields. A concentration of 150 µM tyrosine enhanced L-DOPA content by 12.75-fold and boosted biomass. It also knowingly raised key antioxidant enzyme activities. The method showed far superior to using polyamines. These results offer a viable commercial-scale alternative to synthetic L-DOPA production (Parthasarathy et al., 2025). Another study optimized the *in vitro* culture of the endangered *Verbascum bugulifolium* by improving chlorosis with a potassium phosphate-enriched MS medium (MS+PP). Polyamines like putrescine and spermidine increased germination rates and leaf production, but usually reduced root formation while supporting elongation. A high concentration of putrescine (80 mg L^−1^) tempted lateral roots from leaf explants. Though the MS+PP medium boosted root phenolic content, the addition of putrescine reduced these valuable compounds. The developed protocols support the species conservation and potential pharmaceutical use (Kıymaz and Acemi, 2025).

#### 4.3.2 ROS inducers

**Figure 1:**
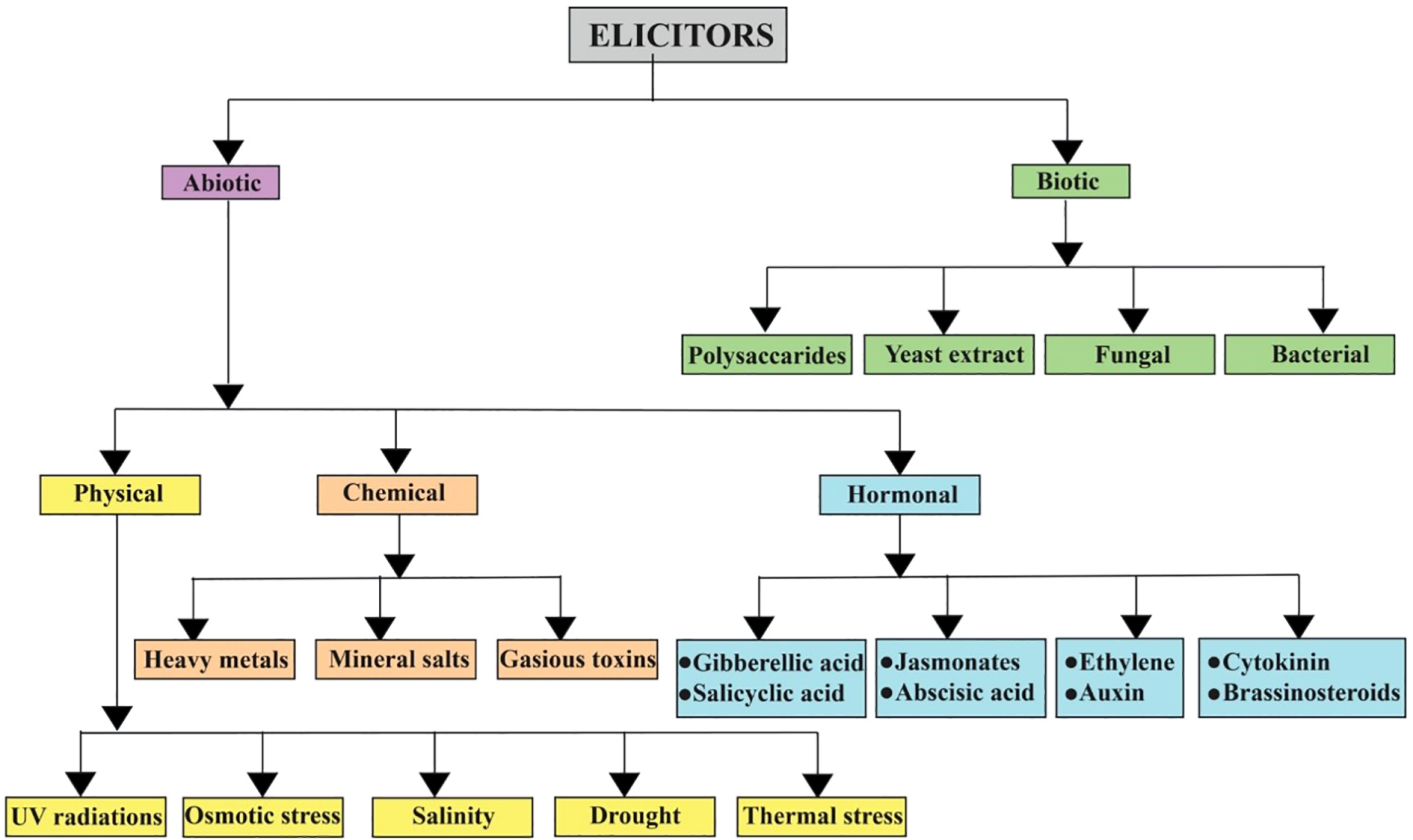
A generalized classification of biotic and abiotic elicitors- highlighting their significance in medicinal plants.

It has been reported that HHT shows substantial therapeutic potential for the treatment of acute myeloid leukemia. Although HHT affects protein synthesis inhibition and apoptosis induction, its accurate anti-leukemic mechanisms remain uncertain. Recent clinical studies have shown the efficacy of HHT combined with cytarabine and etoposide in leukemia induction therapy (J. Chen et al., 2017). Moreover, ETP-resistant cancer cells show sensitivity to HHT, signifying a lack of co-resistance between the two drugs (Zhang et al., 2019). Hence, HHT and etoposide exert synergistic cytotoxicity in acute myeloid leukemia cells through a reactive oxygen species (ROS) dependent mechanism, with disruption of the thioredoxin antioxidant defense system playing an important role (Abdi et al., 2025). found that treating lemon balm with elicitors, such as hydrogen peroxide, hydrogen sulfide, or potassium phosphite, efficiently increases its medicinal value. All treatments improved photosynthetic pigments and boosted the production of the valuable compound rosmarinic acid (RA). This was achieved by elevating key biosynthetic genes and enzyme activity, providing a safe method to improve the plant’s bioactive compound content. Just as the elicitors increased biosynthetic genes and improved enzyme activity to increase RA yield in lemon balm, a similar method could be useful to *Cephalotaxus*. Similarly (Hao et al., 2014), investigated the relationship between salicylic acid and hydrogen peroxide (H_2_O_2_) in the production of RA in *Salvia miltiorrhiza* cell cultures. It found that SA knowingly increases the production of H_2_O_2_, which acts as a key secondary messenger. This H_2_O_2_ signal enhances the activity of the phenylalanine ammonia-lyase (PAL) enzyme, resulting in greater accumulation of RA. The critical role of H_2_O_2_ was confirmed when using inhibitors to block its production or scavenge it; both actions prevented RA accumulation. The results determine that H_2_O_2_ is an essential signaling molecule induced by SA to endorse the synthesis of this valuable secondary metabolite. A generalized classification of elicitors is given in **Figure 1**, and a comparative table of biotic elicitors is given in **Table 2**.

**Table 2:** Comparative efficacy of biotic elicitors in enhancing secondary metabolite production across different plant species and culture systems.

Biotic elicitor	Plant species	Culture system	Elicited compound(s)	Key efficacy/enhancement	Reference
Fungal elicitors					
*Mucor hiemalis*	*Taverniera cuneifolia*	Root cultures	Glycyrrhizic acid (GA)	Maximum GA yield among fungi (4.90 ± 0.10 mg/g)	Awad et al., 2014
*Trichoderma harzianum*	*Panax sikkimensis*	Cell suspension	Ginsenosides, Anthocyanins	Greatest induction of ginsenosides and anthocyanins	Biswas et al., 2018
Bacterial elicitors					
*Rhizobium leguminosarum*	*Taverniera cuneifolia*	Root cultures	Glycyrrhizic acid (GA)	Peak GA production among all tested elicitors (6.37 ± 0.41 mg/g)	Awad et al., 2014
*Escherichia coli*	*Helicteres isora* L.	Suspension culture	Diosgenin	Promoted diosgenin production (culture filtrate used)	Shaikh et al., 2020
Polysaccharide elicitors					
Yeast Extract	*Lippia alba*	*In vitro* plants	Essential oils, Bioactive metabolites	Triggered a single novel metabolite; improved growth traits and essential oil content	Silva-Santos et al., 2023
Chitosan	*Withania somnifera*	Hairy root	Withaferin-A	Employed to enhance Withaferin-A production	Thilip et al., 2019
Yeast-derived Polysaccharides (200 mg/L)	*Fagopyrum tataricum*	Hairy root	Rutin, Quercetin	Induced phenylpropanoid pathway; flavonoid content increased up to 2.1-fold	Halder et al., 2019

## 5 Signal transduction pathways

**Figure 2:**
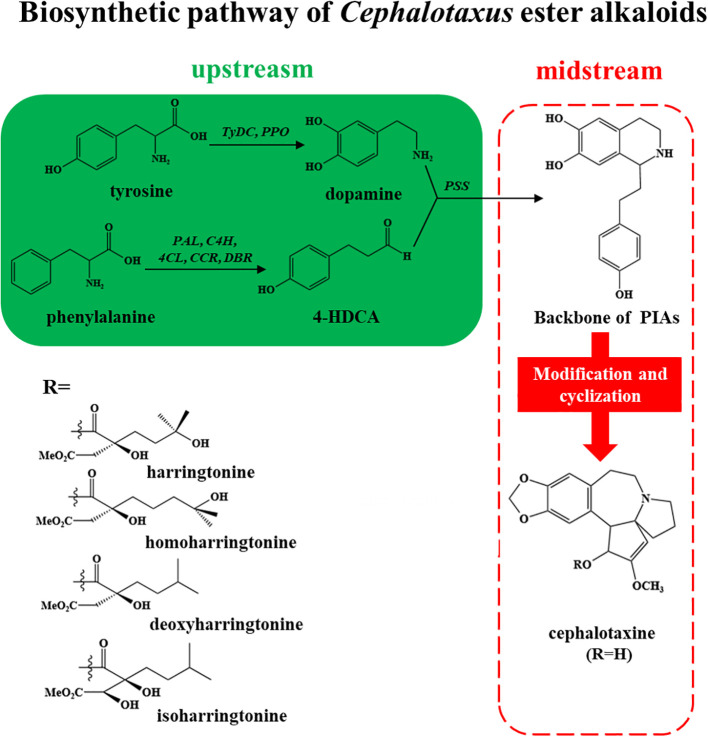
A generalized schematic biosynthetic pathway of *Cephalotaxus-*derived alkaloids.

**Figure 3:**
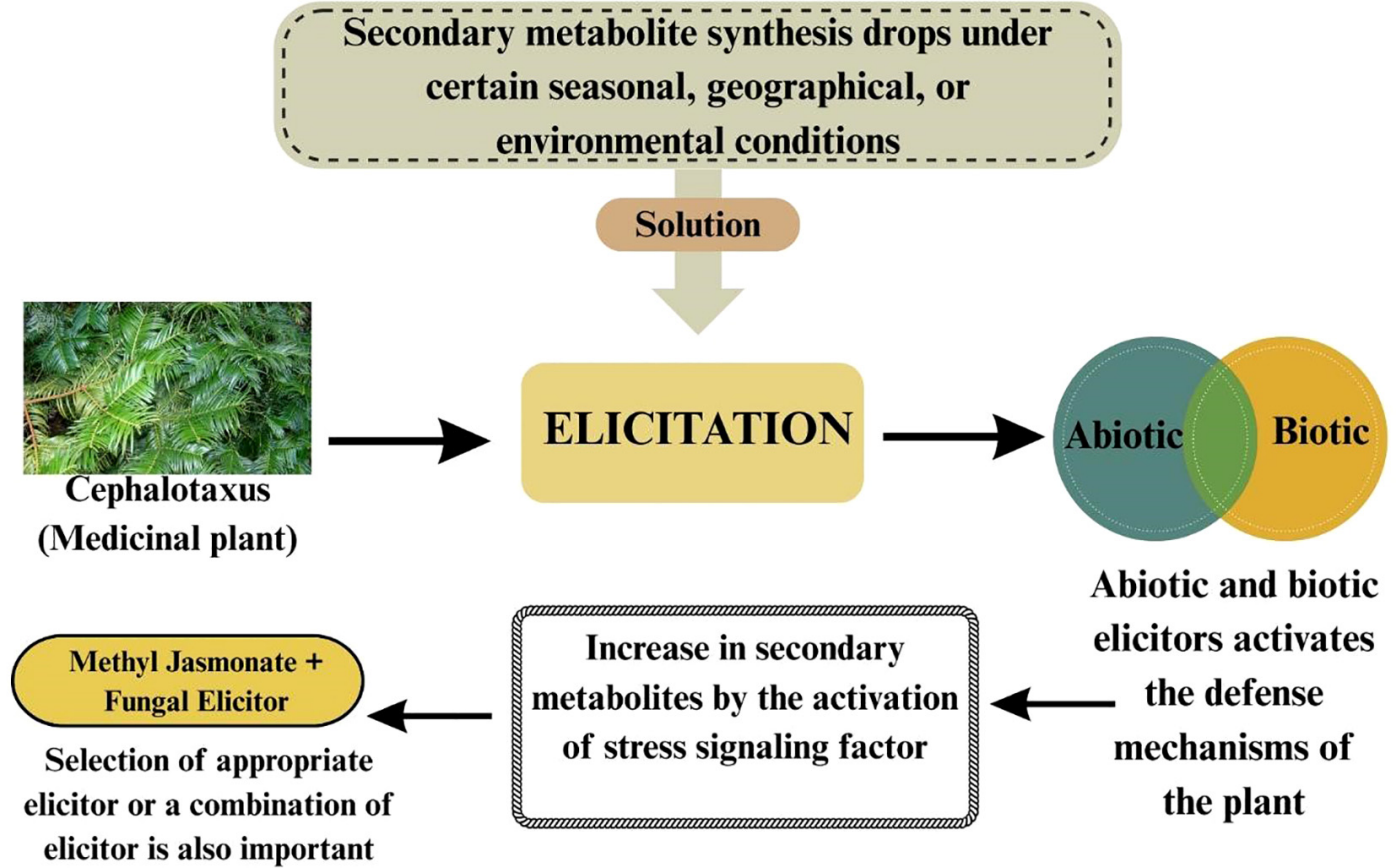
Effect of abiotic and biotic elicitors on secondary metabolite synthesis in *Cephalotaxus* (a medicinal plant).

Plants coordinate throughout the body via systemic signaling, which triggers defense responses and adaptation in plants to withstand stress conditions. Systemic acquired acclimation (SAA) is mediated by key signaling pathways, which include ROS, electric and hydraulic waves, where ROS plays a significant role and is shaped by hormones such as JA, SA, ABA, and ethylene. Research in *Arabidopsis thaliana* showed that SA and JA have contrasting impacts on the ROS wave, where SA strengthens it while JA dampens the ROS wave during response to high light stress. It also elucidated that ABA and ethylene have crucial roles in modulating the ROS wave in whole-plant stress signaling. Furthermore, Nonexpressor of Pathogenesis Related protein 1 (NPR1) is vital for systemic ROS accumulation and enabling SAA under high light stress, reflecting the intricate hormonal crosstalk in plant-wide stress signaling (Myers et al., 2023). However, the MAPK cascades are another critical signaling system that can be influenced by or interact with the same hormonal signals (JA, SA, etc.). MAPK cascade, which is a crucial signaling pathway allowing plants to detect and respond to various biotic and abiotic stress conditions, is regulated by MAPKKK (MAP3Ks) genes, positioned at the top of the cascade. These are the upstream components that initiate the signaling cascade by detecting external stimuli and relaying them to downstream proteins like MAPKKs and MAPKs, executing targeted responses. Despite their crucial role in plants’ development and stress signaling, the specific pathways and downstream MAPKKs or MAPKs regulation are still not well characterized (Xie et al., 2023). A schematic biosynthesis of *Cephalotaxus* producing alkaloids is given in **Figure 2** and a generalized pathway of producing secondary metabolites using elicitors is given in **Figure 3**.

## 6 Synergistic application of combined elicitors

The individual application of elicitors enhances the SM production, but the central thesis of this review is that their *synergistic combination* offers a more powerful and promising strategy. This approach provides leverage of signaling crosstalk, leading to a greater upregulation of biosynthetic pathways.

The most compelling evidence within the scope of medicinal plants like *Cephalotaxus* comes from the work of Li (2014), who demonstrated that the combined application of sodium fluoride (NaF) and methyl jasmonate (MeJA) in *C. mannii* cell cultures acted synergistically. While NaF primarily boosted primary metabolism (enhanced G6PDH activity) and MeJA targeted secondary metabolism (increased PAL activity), their combination resulted in a 4.8-fold increase in harringtonine yield and the distinct production of homoharringtonine (HHT). This case underscores how elicitors with complementary mechanisms can orchestrate a more robust metabolic response. Similar synergistic effects are documented in other species. For instance, in *Artemisia annua* cell suspensions, the combination of sorbitol (an osmotic stress inducer) and coronatine (a jasmonate mimic) synergistically upregulated artemisinin biosynthetic genes and significantly boosted artemisinin content across all tested doses (Salehi et al., 2019). The success of such combinations hinges on several factors, including the careful selection of elicitor types, their dosage, and crucially, their timing (e.g., sequential versus concurrent application) to exploit positive signaling interactions, such as those between JA and ROS pathways (Humbal and Pathak, 2023; Zhang et al., 2019).

Therefore, dedicating research to understanding the mechanisms behind elicitor synergy—particularly the crosstalk between MAPK cascades, hormonal signals (JA, SA, ABA), and ROS waves—is essential to designing highly effective elicitation protocols for the scalable production of valuable compounds like harringtonine and HHT.

## 7 Therapeutic potential of alkaloids in plant species: a case study

*In vitro* studies on *Cephalotaxus* provide further evidence for analyzing SMs production, particularly the biosynthesis of pharmacologically important alkaloids, such as HHT. These studies typically involve cell suspension cultures, hairy root systems, and callus cultures, which give unique advantages for scalable compound extraction (Yong-Gonzalez et al., 2025). investigated the resistance profile of HHT and its derivatives, deoxyharringtonine (DHT), deoxyhomoharringtonine (DHHT), and Bis (demethyl)-deoxyharringtonine (BDHT), in leukemia cell lines and primary patient samples. The HHT was found to be a substrate for MDR1-mediated efflux. It leads to resistance in K562 and MES-SA/MX2 cells, and its derivatives adapted at the acyl chain overcome this resistance. Particularly, primary leukemia cells remained highly sensitive to HHT and its derivatives, showing low nanomolar strength. Automatically, HHT and DHHT induced caspase-3-dependent apoptosis. The study also recognized comparative sensitivities to other drug classes, including CDC7 kinase inhibitors and topoisomerase inhibitors. This research highlights the potential of HHT derivatives to bypass MDR1 resistance, further supporting clinical assessment in AML and other types of cancers (Yang et al., 2022). studied cephalotaxine-type alkaloids, such as HHT, show durable antileukemic properties. This research developed an enantioselective hydrogenation method for β-substituted itaconic acid monoesters using chiral Ru [DTBM-SegPhos], allowing the semisynthesis of novel cephalotaxine byproducts with chiral 2′-substituted-succinic acid 4-monomethyl ester side chains. The initial structure-activity relationship (SAR) studies identified compound 10b, which confirmed potent antileukemic effects and broad anticancer activity across multiple cancer cell lines. The research delivers a well-organized asymmetric hydrogenation approach for producing cephalotaxine derivatives, showing promising candidates for additional anticancer drug development.

**Figure 4:**
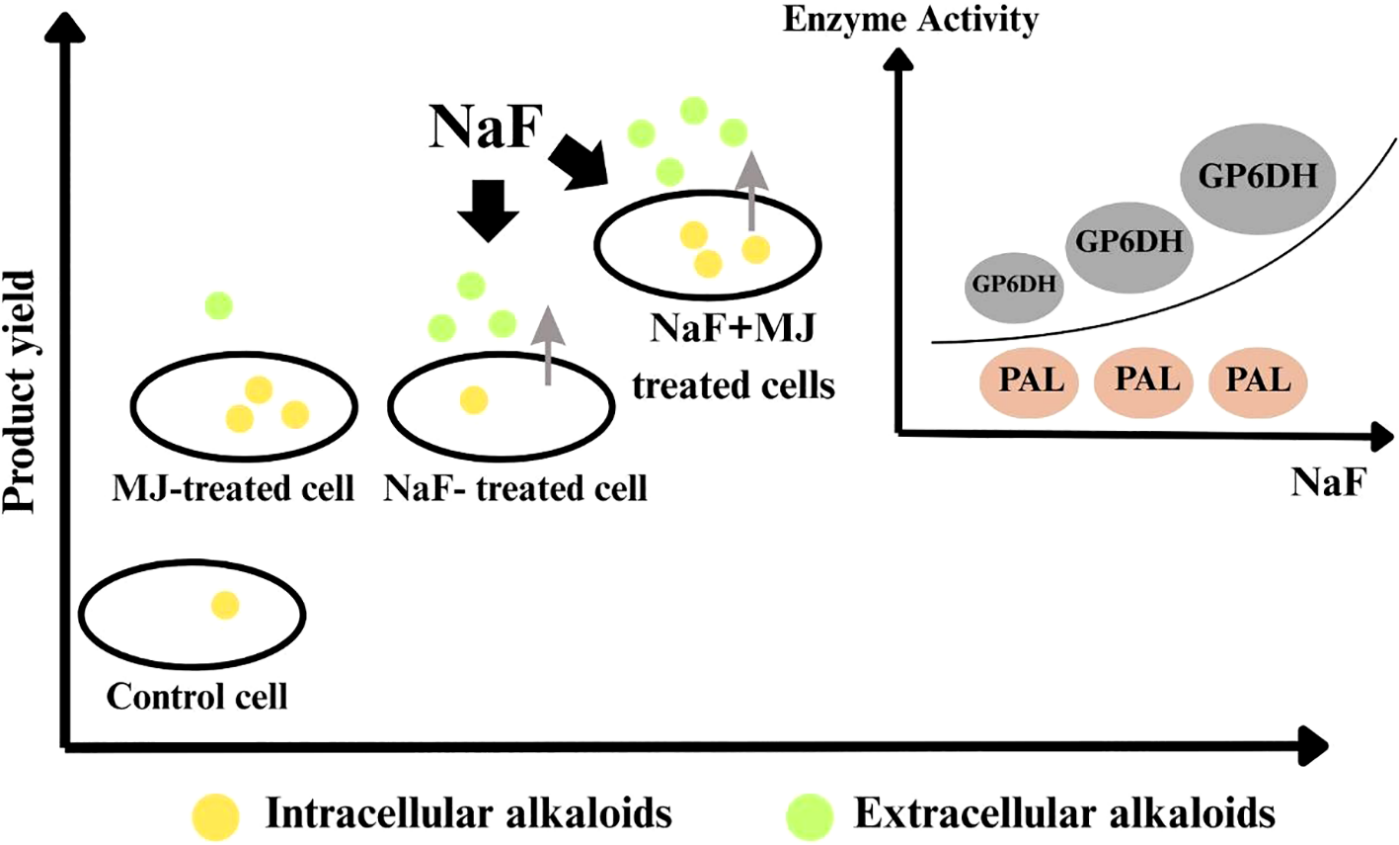
illustrates that the combination of NaF (sodium fluoride) and MJ (methyl jasmonate) enhances the production and secretion of cephalotaxine in a cell culture system. MJ increases alkaloid production inside cells, while NaF boosts both production and secretion. Together, both MJ and NaF yield a high cephalotaxine. NaF enhances the activity of GPDH (key for production/secretion) but has a minute effect on PAL (Li, 2014).

The combination of HHT with venetoclax (VEN) and azacitidine reveals greater efficiency over VEN-azacitidine alone in relapsed/refractory AML, improving response rates and survival. Mechanistically, HHT augments VEN sensitivity by repressing anti-apoptotic proteins (MCL-1/BCL-xL), increasing ROS production, and disrupting mitochondrial membrane potential. Moreover, HHT decreases fatty acid uptake, additionally weakening leukemia cell survival. Preclinical studies approve strong synergetic effects, even in resistant AML cells and MSC-protected environments, by conquering proliferation and triggering cell cycle arrest. These studies propose that HHT augments VEN-based therapy, showing a promising plan to overcome resistance and improve results in high-risk AML patients (Yin et al., 2024).

(Li, 2014) examined how sodium fluoride (NaF) and methyl jasmonate (MJ) affect cephalotaxine production in *Cephalotaxus mannii* cell cultures. NaF boosted G6PDH activity but not PAL or phenols, while MJ augmented PAL activity and phenols without disturbing G6PDH. When combined with NaF+MJ, they synergistically enhanced cephalotaxine production, yielding 7.245 mg/L harringtonine, 4.8 times higher than the control and distinctively producing 0.491 mg/L HHT. NaF+MJ also enhanced secretion, with a 62% release rate versus 78% (NaF alone) and 24% (MJ alone). These results reveal that NaF and MJ together enhance the biosynthesis of cephalotaxines in *C. mannii*, which can be seen “in general” in **Figure 4**.

## 8 Challenges and future perspectives

Elicitors play a vital role in enhancing SMs production, offering substantial potential for plant tissue culture systems. To maximize yields, it is crucial to understand the intricate biosynthetic pathways of plant-derived compounds and their responses to single or combined biotic or abiotic elicitors. Still, inadequate knowledge of vital enzymes, genes, transcription factors, and regulatory switches remains a significant barrier to improving metabolite synthesis (Guru et al., 2022). Plant metabolism is actively engaged, and its activity fluctuates under various conditions. An elicitor’s effect can be strong at one time point but weak or altered at another, making it difficult to capture transient changes or feedback regulation with single time point studies. Furthermore, metabolic flux can change between pathways, so increasing one compound may decrease others due to precursor competition (Wang et al., 2025). Multi-omics revelations can guide genetic improvements in native plant producers, such as using CRISPR/Cas9 to interrupt negative feedback that decreases metabolic pathways. Moreover, nanotechnology-based elicitation shows great potential for enhancing the production of therapeutic plant compounds *in vitro*; however, further research is needed to optimize nanoparticle applications. With increasing demand for plant-derived pharmaceuticals and nutraceuticals, biotechnological strategies, including *in vitro* culture and targeted elicitation, offer feasible solutions to enhance the production of these beneficial bioactive compounds (Karalija et al., 2025). To completely utilize the therapeutic potential of traditional medicinal plants, we must overcome important challenges, including large-scale data management, standardization of methods, dynamic metabolic analysis, and translational validation. Advances such as global genome sequencing projects, standardized omics protocols, and bioactivity assessment tools will help bridge the gap between traditional plants and well-studied model crops. By addressing these hurdles, we can attain accurate bioengineering and optimization of medicinal species for human health applications (Wang et al., 2025).

## 9 Conclusion

This review establishes elicitor-mediated strategies as a transformative approach for enhancing secondary metabolite production in medicinal plants, using *Cephalotaxus* and its anti-leukemia alkaloids as a key model. It demonstrates that the synergistic application of combined elicitors, such as NaF and MeJA, is particularly effective, surpassing individual treatments by activating complementary biosynthetic pathways. While challenges in metabolic flux and process optimization remain, integrating elicitation with emerging biotechnologies like multi-omics, CRISPR-based engineering, and nanotechnology offers a powerful, sustainable framework for the scalable production of high-value plant-derived pharmaceuticals, bridging the gap between traditional knowledge and modern therapeutic demand.
